# Supplementary material for: Antimicrobial and Cytotoxic Activities of the Secondary Metabolites of Endophytic Fungi Isolated from the Medicinal Plant Hyssopus officinalis
Source: Antibiotics (Basel). 2023 Jul 18;12(7):1201. doi: 10.3390/antibiotics12071201 (PMC10376753; doi:10.3390/antibiotics12071201)
Supplement: Supplementary file 1 [file antibiotics-12-01201-s001.zip › antibiotics-2489668-supplementary.pdf]

SUPPLEMENTARY MATERIAL

Antimicrobial and Cytotoxic Activities of the Secondary Metabolites of Endophytic Fungi Isolated from the Medicinal Plant *Hyssopus officinalis*

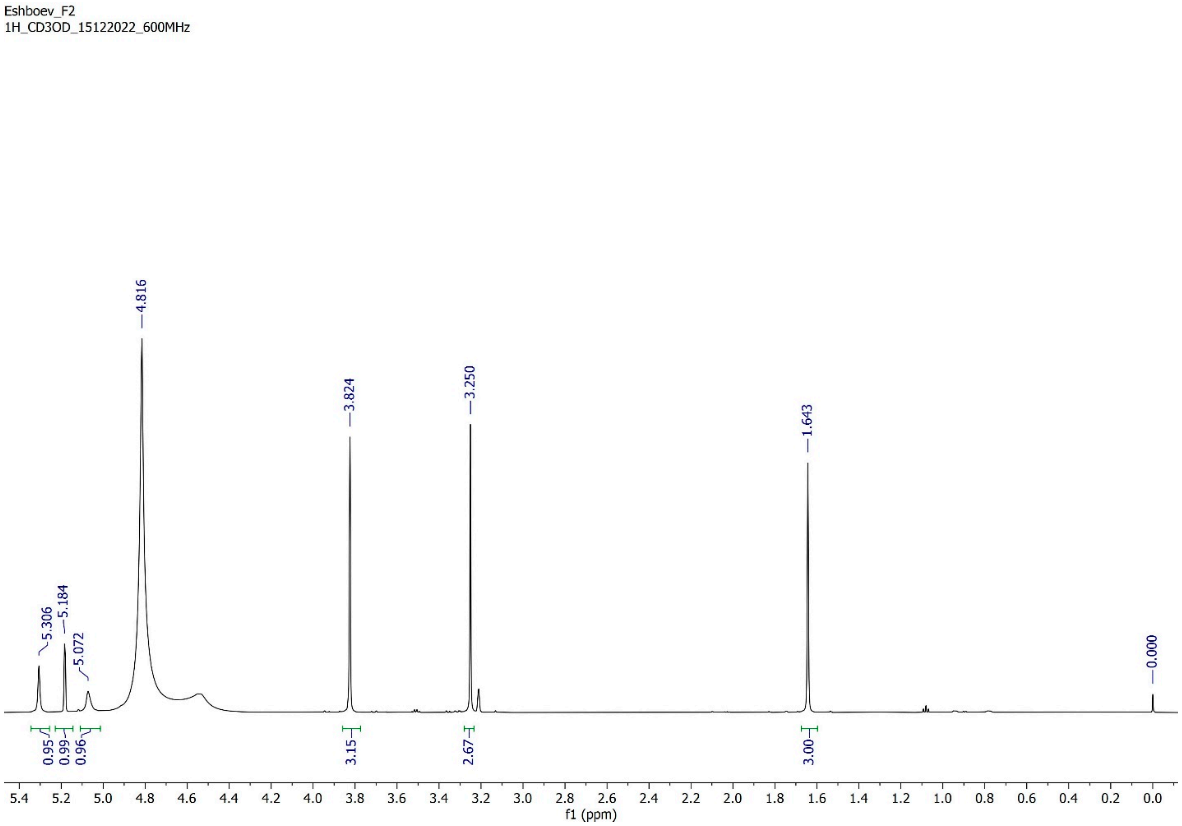

Figure S1. ^{1}H NMR (600 MHz) spectrum of compound **1** in CD_3OD.

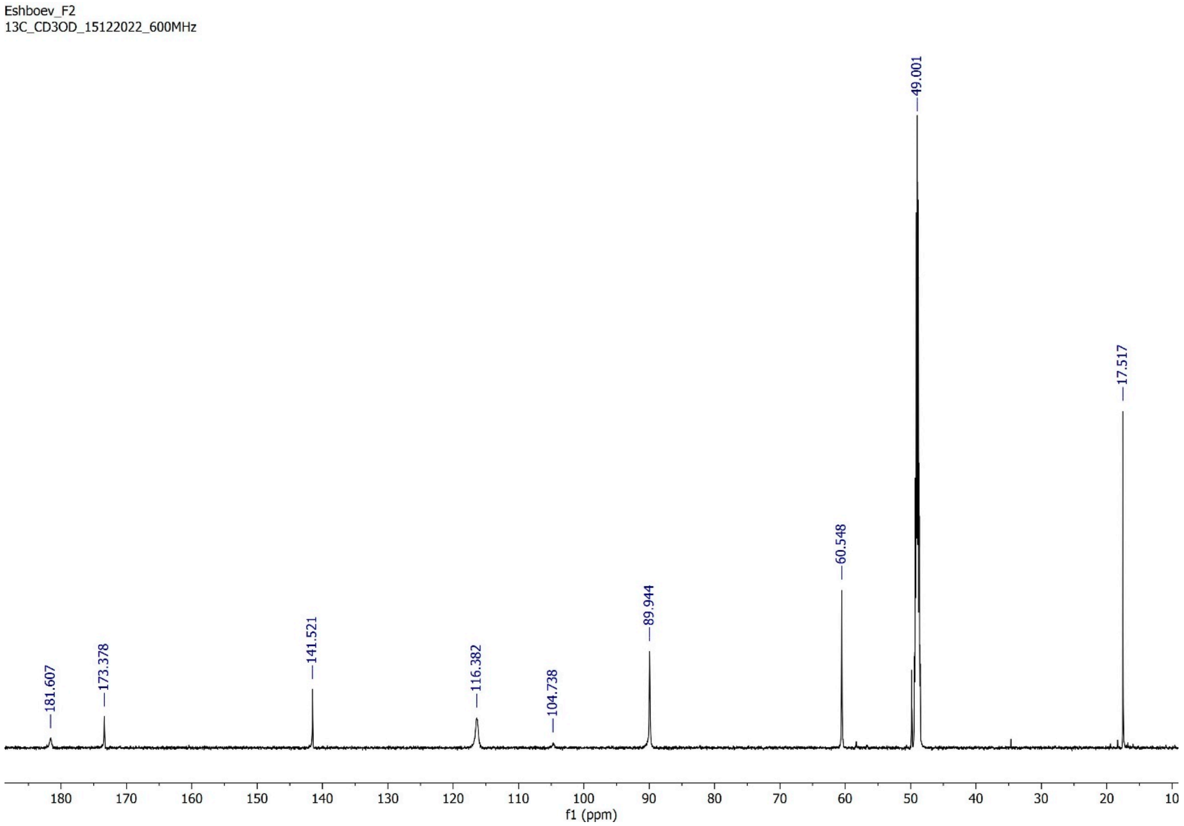

Figure S2. ^{13}C NMR (150 MHz) spectrum of compound **1** in CD_3OD.

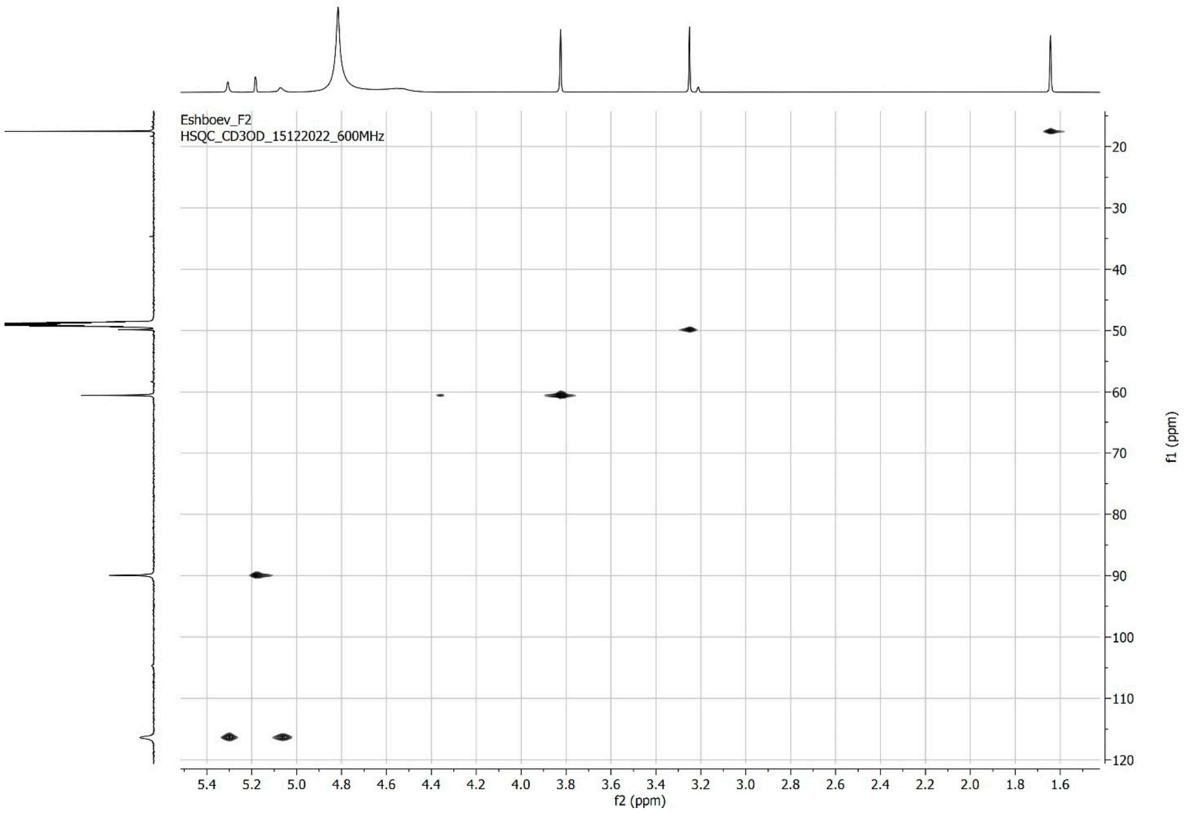

Figure S3. HSQC spectrum of compound **1** in CD_3OD.

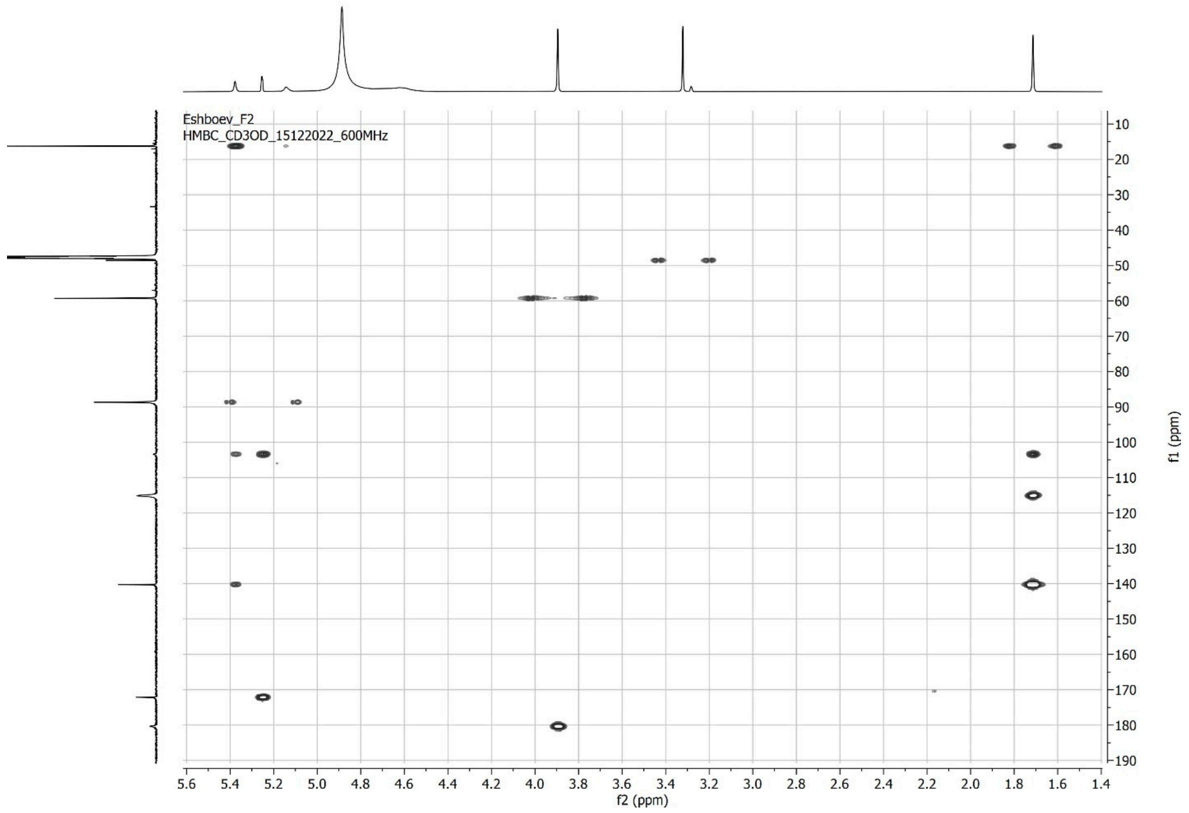

Figure S4. HMBC spectrum of compound **1** in CD_3OD.

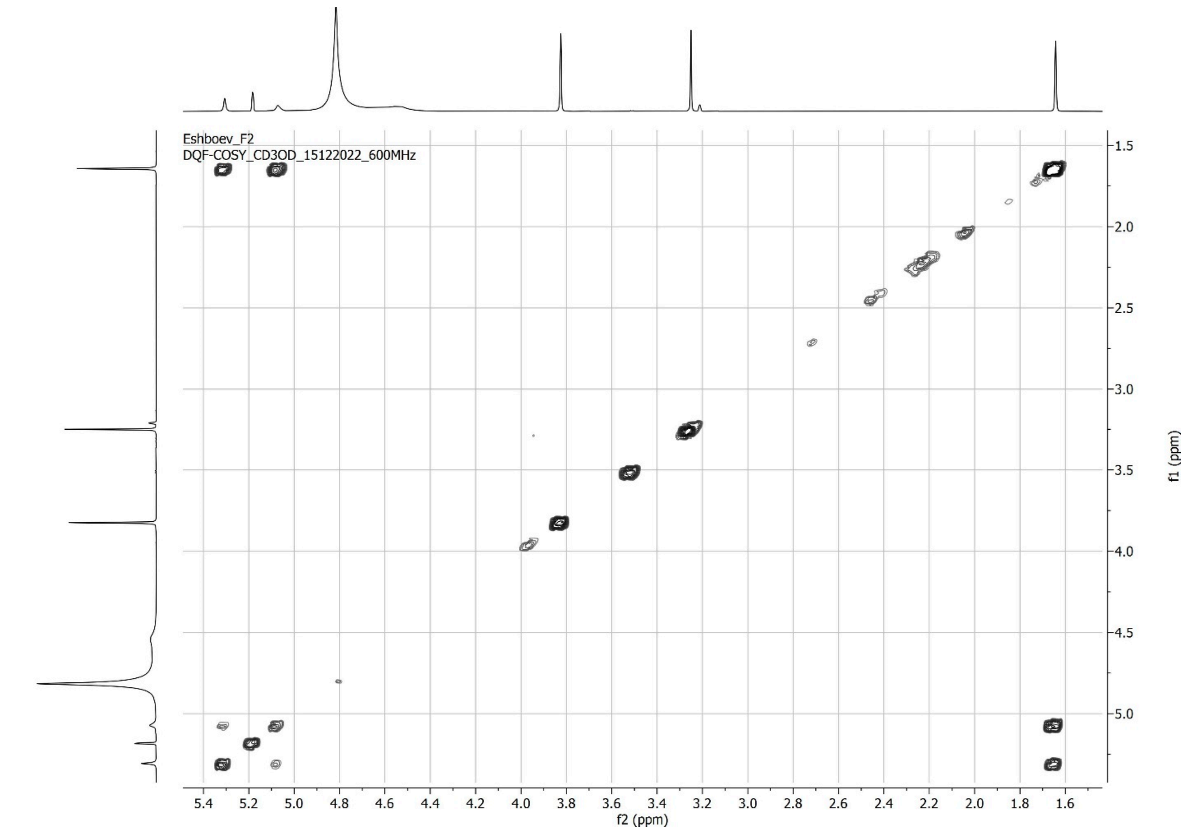

Figure S5. COSY spectrum of compound **1** in CD_3OD.

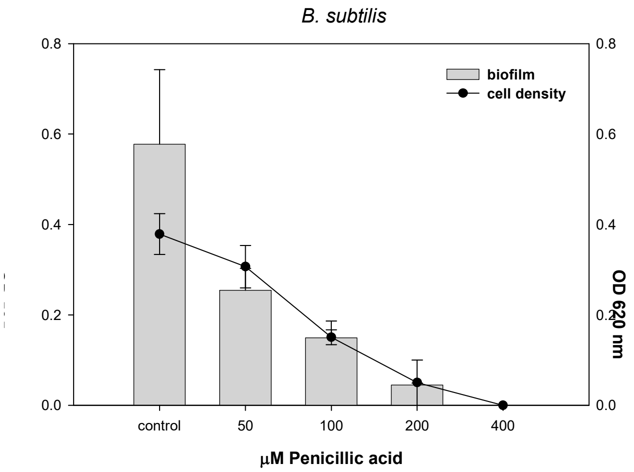

Figure S6. Biofilm formation of *B.subtilis* at different concentrations of penicillic acid.
